# No change in the consumption of thyroid hormones after starting low dose naltrexone (LDN): a quasi-experimental before-after study

**DOI:** 10.1186/s12902-020-00630-4

**Published:** 2020-10-01

**Authors:** Guttorm Raknes, Lars Småbrekke

**Affiliations:** 1 Regional Medicines Information and Pharmacovigilance Centre (RELIS), University Hospital of North Norway, Tromsø, Norway; 2 Raknes Research, Ulset, Norway; 3 Department of Pharmacy, Faculty of Health Sciences, UiT - The arctic university of Norway, Tromsø, Norway

**Keywords:** Endocrinology, Hypothyroidism, Naltrexone, Levothyroxine, Triiodohyronine, Pharmacoepidemiology

## Abstract

**Background:**

Low dose naltrexone (LDN) is reported to have beneficial effects in several autoimmune diseases. The purpose of this study was to examine whether starting LDN was followed by changes in the dispensing of thyroid hormones to patients with hypothyroidism.

**Methods:**

We performed a quasi-experimental before-after study based on the Norwegian Prescription Database. Study participants were identified by using reimbursement codes for hypothyroidism. Cumulative dispensed Defined Daily Doses and the number of users of triiodothyronine (T3) and levothyroxine (LT4) 1 year before and after the first LDN prescription was compared in three groups based on LDN exposure.

**Results:**

We identified 898 patients that met the inclusion criteria. There was no association between starting LDN and the subsequent dispensing of thyroid hormones. If anything, there was a tendency towards increasing LT4 consumption with increasing LDN exposure.

**Conclusion:**

The results of this study do not support claims of efficacy of LDN in hypothyroidism.

## Background

The opioid antagonist naltrexone in low doses (typically < 5 mg/day) is being used off-label against several autoimmune diseases. In a recent review, the authors claim that low dose naltrexone (LDN) has beneficial immunomodulatory effects by acting on the opioid growth factor receptor (OGFr) or as a direct immunomodulating agent, by elevating endogenous opioids, or by affecting cytokine production [1].

A few small randomized clinical trials show promising results in inflammatory bowel disease, multiple sclerosis and chronic pain conditions [1]. Although no studies in thyroid disease have been published, LDN has been proposed as an alternative add-on to regular hypothyroid therapy. Some patients report astounding improvements [2], and there are patients and doctors who claim that LDN may be beneficial in autoimmune thyroid disease, but definitive evidence is lacking [3].

Decades of high dose naltrexone in the treatment of opioid and alcohol addiction indicate that LDN has an excellent safety profile [4], and with increasing awareness among patients, it is highly likely that endocrinologists increasingly will have requests to prescribe LDN.

In 2013, the number of users of LDN in Norway increased from almost zero to 0.3% of the population within few months following a TV documentary [5]. This surge in LDN use could be considered a natural experiment that make quasi-experimental pharmacoepidemiological studies possible. We seized the opportunity to examine whether there were changes in the dispensing of thyroid hormones in selected patients following the initiation of LDN therapy. If LDN is actually efficacious in hypothyroidism, it is plausible that it would result in changes in the use of triiodothyronine (T3) and/or levothyroxine (LT4).

## Methods

This was a controlled before-after study based on data from the Norwegian Prescription Database (NorPD). The NorPD contains information on prescriptions dispensed to all Norwegian residents [6]. For a fee, NorPD used reimbursement codes for hypothyroidism (International Classification of Disease (ICD-10) code E03 and International Classification of Primary Care (ICPC-2) code T86) to identify patients in the database according to our specifications. We received an encrypted data file allowing us to follow individual patients without knowing their identity. The database does not contain any other clinical information related to thyroid disease than the prescribing of thyroid hormones. To increase specificity, we only included patients that collected at least two prescriptions containing reimbursement codes for hypothyroidism in 2009 and 2010, and had collected ≥1 LDN prescriptions in 2013. We defined the first prescription in 2013 as index data, and stratified patients in three groups according to LDN exposure in the 2 years after index date: LDN × 1 (controls, collected LDN once), LDN × 2–3 (collected LDN twice or thrice) and LDN × 4+ (persistent users, collected LDN > 3 times). We have previously used the same stratification in other studies [7–9].

Outcomes were differences between the cumulative collected amounts of Defined Daily Doses (DDDs) and the number of users of levothyroxine (LT4) and triiodothyronine (T3). One DDD LT4 equals 150 μg, and one DDD triiodothyroxine is 60 μg. We calculated the difference for each patient by subtracting the number of collected DDDs of T3 and LT4 year following the first LDN prescription from the number of DDDs in the year preceding the first LDN prescription. All index dates were in 2013, and the total observation time was 2 years for all participants. The first observation date was theoretically January 1, 2012, and the last observation date was December 31, 2014.

### Statistical methods

The study size depended on the number of patients in NorPD meeting our inclusion criteria. We used SPSS 25 and Excel 2013 for data management and statistical analysis, and analyzed all data on individual level. We used a pairwise two-sided *t*-test to investigate the mean changes in the sum of the DDDs per patient in each group for all examined medicines. In addition, we calculated 95% confidence intervals (CI) for the difference of means. Change in the number of users was expressed as the proportion of each group, together with the 95% CI for the difference of the proportion (in % points) [10]. Difference-in-difference of DDDs and the proportion of users (in % points) with 95% CI were calculated.

In addition, we conducted an ANCOVA to compare differences in T3 and LT4 dispensing before and after index date while adjusting for sex, age in 2013, and the number of prescriptions (all prescription medicines) collected 1 year before index date.

## Results

We identified 898 patients who met the inclusion criteria. Baseline data are given in Table 1. There were negligible differences between groups in age, proportion females and the cumulative number of prescriptions of all medicines per person 1 year before index date.

**Table 1:** Baseline data

	LDN × 1		LDN × 2–3		LDN × 4+	
**N (%)**	260	(29.0)	198	(22.0)	440	(49.0)
**Female (%)**	240	(92.3)	178	(89.9)	415	(94.3)
**Age (±SD)**	58.0	(12.2)	55.7	(12.4)	54.7	(13.0)
**Number of prescriptions before LDN** **(all drugs) (±SD)**	32.1	(38.3)	30.8	(32.6)	30.8	(13.4)

Differences in DDD are shown in Table 2. There were no T3 or LT4 DDD differences between groups before or after starting LDN, or within groups before-after. The difference-in-difference between LDN × 1 and LDN × 4+ for LT4 was not significant (6.2 DDD, 95% CI − 11.3 to 23.7, *p* = 0.313), but the dispensed cumulative DDD in LDN × 4+ LT4 dispensing after was almost significantly larger after starting LDN (19.8 DDD, 95% CI − 0.1 to 23.7, *p* = 0.060).

**Table 2:** Average cumulative number of DDDs of thyroid hormones dispensed to patients 1 year before and after the first dispense of LDN. Patients stratified based on the number of LDN dispenses: LDN × 1 (*N* = 260), LDN × 2–3 (*N* = 198) and LDN × 4+ (*N* = 440). LDN: Low dose naltrexone. DDD: Defined daily dose

	Dispensed drugs (DDD)		Difference (DDD)		
	Before	After	Mean	95% CI difference	p
**Levothyroxine (LT4)**					
LDN × 1	225.4	223.1	−2.3	(−15.9 to 11.2)	0.737
LDN × 2–3	245.9	248.9	3.0	(−15.6 to 21.5)	0.754
LDN × 4+	239.0	242.9	3.9	(−7.1 to 14.9)	0.490
**Triiodothyronine (T3)**					
LDN × 1	10.4	10.5	0.1	(−2.8 to 3.1)	0.932
LDN × 2–3	8.1	5.9	−2.2	(−5.1 to 0.7)	0.139
LDN × 4+	11.4	11.1	−0.3	(−2.9 to 2.3)	0.818

There were no differences in the number of users of neither T3 nor LT4, except for a 2.0% points borderline significant reduction in the number of LT4 users in the LDN × 2–3 group (Table 3). There was no difference-in-difference in number of users of LT4 between LDN × 1 and LDN × 4+ (1.2% points, 95% CI − 1,8 to 4,1, *p* = 0.441) or between LDN × 2–3 and LDN × 4+ (2.0% points, 95% CI − 0.5 to 4.5, *p* = 0.113). For the number of T3 users, there was no difference-in-difference between groups, for example LDN × 1 vs. LDN × 4+: 2.4% points, 95% CI − 0.9 to 5.8, *p* = 0.151).

**Table 3:** The number of users of thyroid hormones dispensed 1 year before and after the first dispense of LDN. The groups are stratified based on the number of LDN dispenses: LDN × 1 (*N* = 260), LDN × 2–3 (*N* = 198) and LDN × 4+ (*N* = 440). LDN: Low dose naltrexone

	Number of users				Difference		
	Before		After				
	N	%	N	%	% points	95%CI	***p***
**Levothyroxine (LT4)**							
LDN × 1	243	(93.5)	240	(92.3)	−1.2	(−3.7 to 1.3)	0.366
LDN × 2–3	191	(96.5)	187	(94.4)	−2.0	(−4.0 to −0.1)	0.045
LDN × 4+	420	(95.5)	420	(95.5)	0.0	(−1.5 to 1.5)	1.000
**Triiodothyronine (T3)**							
LDN × 1	28	(10.8)	24	(9.2)	−1.5	(−4.1 to 1.1)	0.248
LDN × 2–3	15	(7.6)	11	(5.6)	−2.0	(−5.1 to 1.1)	0.206
LDN × 4+	51	(11.6)	55	(12.5)	0.9	(−1.2 to 3.0)	0.394

The results of the ANCOVA analyses are given in Additional file 1. Levene’s test and normality checks were carried out and the assumptions met. When adjusting for sex, age and number of prescriptions dispensed before index date in the model, no differences between LDN groups in the change of dispensing was observed for neither T3 (F (2, 892) = 0.53, *p* = 0.588)) nor LT4 (F (2, 892) = 0.246, *p* = 0.782)).

## Discussion

We found no association between starting LDN and changes in the dispensing of thyroid hormones. If anything, there was a tendency towards increasing LT4 consumption with increasing LDN exposure.

There was a reduction in users LT4 in the group with intermediate LDN exposure (LDN × 2–3), but this does not suggest efficacy of LDN in hypothyroidism. There was no significant difference-in-difference between groups. Since the persistent LDN users (LDN × 4+) had the least reduction in the number of LT4 users, this finding does not fit into a dose-response relationship.

NorPD is a reliable data source that contains almost complete information on all prescribing outside institutions in Norway, and the inclusion criteria probably lead to a representative sample of the LDN using hypothyroidism population. The strict inclusion criteria reduced the risk of accidental misdiagnosis and bias due to patients starting thyroid hormones during the observation period. Register-based studies have important limitations, and among the most important is scarce clinical information. The prescribing of T3and LT4 are only proxies to thyroid function, and we cannot rule out possible beneficial or negative effects of LDN not leading to changes in the dosing of thyroid hormones. In addition, the data did not allow any stratification between different types of hypothyroidism, and it was not possible to adjust for body weight. It is possible that the use of large amounts of thyroid hormones in obese patients could cancel data from leaner patients. We do not believe this was a major source of bias, since the outcome was pairwise *differences* in prescribing of T3 and LT4, not total cumulative use.

Quasi-experimental studies have limitations due to non-random assignment, and in this study, we did not include a control group that was unexposed to LDN. Bias in the inclusion of patients, temporal factors such as natural course of disease and regression to the mean are other problems associated with before-after studies [11].

The negative findings in this study fit into the mixed results of clinical and pharmacoepidemiological studies on LDN in autoimmune disease [1, 8, 9].

## Conclusions

The initiation of LDN therapy was not followed by changes in the use of thyroid hormones. The results of this study do not support claims of efficacy of LDN in hypothyroidism.

## Supplementary information

**Additional file 1:.** ANCOVA analyses

## Data Availability

Restrictions apply to the availability of data generated or analyzed during this study to preserve patient confidentiality or because they were used under license. The corresponding author will on request detail the restrictions and any conditions under which access to some data may be provided. Data is available from the Norwegian Prescription Database (NorPD) for researchers who after application meet the criteria for access to prescription data. This includes Ethics Committee evaluation and a fee will be charged. For further details, please see https://www.fhi.no/en/hn/health-registries/norpd/Access-data-norpd/. We did not have any special privileges accessing the data, we obtained the data through the same application and fee payment process as outlined here. Our approvals do not allow the publication of raw data used in this study. In accordance with NorPD’s requirements, we had to delete all original data by 31 December 2018.

## Abbreviations

DDD: Defined Daily Dose
ICD: International classification of disease
ICPC: International Classification of Primary Care
LDN: Low dose naltrexone
NorPD: The Norwegian Prescription Database
OGFr: Opioid growth factor receptor
T3: Triiodothyronine
LT4: Levothyroxine
