# Supplementary material for: No change in the consumption of thyroid hormones after starting low dose naltrexone (LDN): a quasi-experimental before-after study
Source: BMC Endocr Disord. 2020 Oct 1;20:151. doi: 10.1186/s12902-020-00630-4 (PMC7528597; doi:10.1186/s12902-020-00630-4)
Supplement: Supplementary file 1 — Additional file 1:. ANCOVA analyses [file 12902_2020_630_MOESM1_ESM.pdf]

Additional file 1: ANCOVA analyses

One-Way ANCOVA analyzing differences between three groups (LDN group) with different low dose naltrexone (LDN) exposure. Controlling for sex, age in 2013 and number of all dispenses (Presc.) one year preceding index date.

a) Average change in triiodothyronine (T3) dispensing one year before vs one year after the first LDN dispense.

Source	Type III Sum of Squares	df	Mean Square	F	Sig.
Corrected Model	1806.8	5	361.4	0.567	0.726
Intercept	22.2	1	22.2	0.035	0.852
Presc.	916.1	1	916.1	1.436	0.231
Age 2013	313.3	1	313.3	0.491	0.484
Sex	17.8	1	17.8	0.028	0.867
LDN group	677.1	2	338.5	0.531	0.588
Error	568982.1	892	637.9		
Total	571105.6	898			
Corrected Total	570788.9	897			

b) Average change in levothyroxine (T4) dispensing one year before vs one year after the first LDN dispense.

Source	Type III Sum of Squares	df	Mean Square	F	Sig.
Corrected Model	86154.9[a]	5	17231.0	1.207	0.304
Intercept	20827.4	1	20827.4	1.459	0.227
Presc.	49780.4	1	49780.4	3.488	0.062
Age 2013	1534.2	1	1534.2	0.108	0.743
Sex	27469.9	1	27469.9	1.925	0.166
LDN group	7033.2	2	3516.6	0.246	0.782
Error	12729273.9	892	14270.5		
Total	12818607.3	898			
Corrected Total	12815428.8	897			
